# A Simulated Microgravity Environment Causes a Sustained Defect in Epithelial Barrier Function

**DOI:** 10.1038/s41598-019-53862-3

**Published:** 2019-11-26

**Authors:** Rocio Alvarez, Cheryl A. Stork, Anica Sayoc-Becerra, Ronald R. Marchelletta, G. Kim Prisk, Declan F. McCole

**Affiliations:** 1 Division of Biomedical Sciences, University of California, Riverside, Riverside, CA 92521 USA; 2 Department of Medicine, University of California, San Diego, La Jolla, CA 92093 USA; 3 Department of Radiology, University of California, San Diego, La Jolla, CA 92093 USA; 4 Present Address: Cedars-Sinai Medical Center, 8700 Beverly Blvd, Los Angeles, CA 90048 USA; 5 Present Address: Johnson & Johnson Research Laboratories, Janssen Pharmaceuticals Inc., La Jolla, CA 92093 USA

**Keywords:** Tight junctions, Gastrointestinal models

## Abstract

Intestinal epithelial cell (IEC) junctions constitute a robust barrier to invasion by viruses, bacteria and exposure to ingested agents. Previous studies showed that microgravity compromises the human immune system and increases enteropathogen virulence. However, the effects of microgravity on epithelial barrier function are poorly understood. The aims of this study were to identify if simulated microgravity alters intestinal epithelial barrier function (permeability), and susceptibility to barrier-disrupting agents. IECs (HT-29.cl19a) were cultured on microcarrier beads in simulated microgravity using a rotating wall vessel (RWV) for 18 days prior to seeding on semipermeable supports to measure ion flux (transepithelial electrical resistance (TER)) and FITC-dextran (FD4) permeability over 14 days. RWV cells showed delayed apical junction localization of the tight junction proteins, occludin and ZO-1. The alcohol metabolite, acetaldehyde, significantly decreased TER and reduced junctional ZO-1 localization, while increasing FD4 permeability in RWV cells compared with static, motion and flask control cells. In conclusion, simulated microgravity induced an underlying and sustained susceptibility to epithelial barrier disruption upon removal from the microgravity environment. This has implications for gastrointestinal homeostasis of astronauts in space, as well as their capability to withstand the effects of agents that compromise intestinal epithelial barrier function following return to Earth.

## Introduction

A microgravity environment, such as that encountered in spaceflight, has been demonstrated to affect basic cellular function in complex biological organisms and in cells. Genetic, biochemical and morphological parameters have all been shown to be modulated by a microgravity environment in multiple cell types^1–6^. A major technical resource in the study of the biological effects of reduced microgravity has been the development of the rotating wall vessel (RWV). The RWV is a rotating bioreactor that maintains cells in a controlled rotation environment where randomization of gravitational vectors creates a low shear simulated microgravity fluid environment. This system serves to model the impact(s) of microgravity or weightlessness on *in vitro* cultured cells. In addition, since the RWV environment is mixed by gentle rotation, lacks an air-fluid interface despite efficient oxygenation, and maintains laminar fluid flow, it avoids the large shear stress caused by turbulent flow^7–12^. Since the RWV minimizes the impact of gravitational force, it permits study of the effects of weight, or lack of weight in this case, on biological systems. Consequently, the environment could most accurately be referred to as “near weightlessness”^13^. One important clarification is that because microgravity simulation experiments can only change the influence of the Earth gravity vector and not its magnitude, true microgravity cannot be fully accomplished with a mechanical simulator^14^. Therefore, a ground-based simulator such as a RWV has the capacity to model the perception of low-shear microgravity by creating a functional weightlessness as perceived by the organism or cell being investigated^11,13^.

Previous studies investigating the response of epithelial cells to reduced or simulated microgravity identified that HT-29.cl19a colonic epithelial carcinoma cell line clones were capable of forming attachments to extracellular matrices following culture in a RWV as efficiently as cultures at normal G^15,16^. This is important in that it suggests that reduced gravity does not affect the ability of intestinal epithelial cells (IEC) to attach to basement membrane proteins, a fundamental step in the formation of a monolayer. Elegant studies by Honer zu Bentrup *et al*.^17^, and Drummond *et al*.^18^, confirmed that HT-29.cl19a and Caco-2 IECs form monolayers when cultured on microcarrier beads, form distinct tight junction and adherens junction complexes and exhibit different patterns of gene expression when cultured in 3-D structures *vs*. conventional 2-D culture in culture plates (non-polarized). Of the genes upregulated in 3-D cultures, many were associated with epithelial differentiation^17,18^. This aligns with earlier findings from studies in renal epithelial cells cultured in RWV or on the space shuttle, that showed dramatic changes in morphology that more resembled epithelial cells *in vivo* compared with 2-D culture, and in the expression of many gene groups including those associated with transcription factors, signaling proteins and cytoskeletal proteins^6,19,20^. Moreover, a clear morphological consequence of altered gravity was shown as cells cultured in the RWV, and to a greater extent on the space shuttle, exhibited increased number and size of microvilli compared with control cells cultured on Earth at normal G. This is an intriguing observation given that epithelial cell culture on Earth is associated with reduced numbers of microvilli compared to epithelial cells *in vivo*^15,19^.

Another striking observation that was made in astronauts post-flight was that they exhibit immunosuppression of multiple immune cell subtypes^21–27^. In addition, the suppressive effects of space flight on immune function are compounded as a risk factor for long term habitation in space by evidence from Wilson *et al*.^28^, showing increased virulence of a foodborne bacterial pathogen, *Salmonella* typhimurium after time spent in space. This was complemented by ground-based studies demonstrating altered bacterial virulence or adherence by either bacterial or epithelial cell culture respectively in the simulated microgravity environment of the RWV^17,18,29,30^.

This prompted our interest in understanding how the barrier properties of the single layer of epithelial cells that line the gastrointestinal tract are affected by microgravity. The barrier function of the intestinal epithelium is critical for the maintenance of intestinal homeostasis and when disrupted, can lead to increased permeability to bacterial products, antigens and precipitate inappropriate inflammatory responses. This can greatly increase the risk of infections, and chronic inflammatory conditions including inflammatory bowel disease, celiac disease, Type 1 diabetes and liver disease^31–34^. Of significance is that these chronic conditions exhibit an increase in intestinal permeability prior to the onset of inflammation as shown in animal models and patient studies^35–37^. Therefore, we set out to assess if exposure of intestinal epithelial cells (IEC) to a simulated microgravity environment resulted in a decrease in barrier function and/or increased susceptibility of the barrier following challenge with an agent capable of compromising the barrier. The permeability-inducing agent we chose to investigate was the alcohol metabolite, acetaldehyde. Alcohol increases gastrointestinal macromolecule permeability in normal subjects, in patients with alcoholic liver disease (ALD) and also in animal models^38–41^. Ingested alcohol undergoes a series of metabolic steps to facilitate its elimination with the generation of acetaldehyde representing the primary, and most toxic, ethanol metabolite. In the gastrointestinal tract, luminal acetaldehyde production can occur via the activity of cytochrome P450 2E1 or alcohol dehydrogenase from the mucosal epithelium or bacterial sources respectively^42,43^. Studies in rats demonstrated that chronic ethanol administration resulted in high concentrations of acetaldehyde in the colonic lumen. Of note, antibiotic treatment partially attenuated this increase, thus indicating a detectable role for gut bacteria in acetaldehyde generation^43^. With respect to pathologic consequences of ethanol ingestion and metabolism, evidence from *in vivo* and *in vitro* studies indicated that acetaldehyde elevates intestinal and cultured IEC permeability to endotoxin or other permeability markers^44–46^. Epithelial barrier function is principally regulated by apical tight junctions^47–49^. Mechanistic studies have demonstrated that increases in paracellular permeability caused by ethanol or acetaldehyde exposure are associated with redistribution of tight junction proteins such as occludin and zona occludens-1 (ZO-1), from the intercellular junctions into intracellular compartments^50–52^. Colocalization of ZO-1, the first identified tight junction plaque protein, and occludin, the first identified tight junction transmembrane protein, at the tight junction is a strong indicator of barrier integrity, and reduction of this membrane colocalization is indicative of a compromised barrier^53–55^. ZO-1 is a scaffold protein that localizes to the apical tight junction where it maintains cell polarity and is essential to the formation of the tight junctional complex^56–58^. While ZO-1 knockout mice are embryonic lethal, the importance of ZO-1 to barrier function was demonstrated by decreased transepithelial electrical resistance in siRNA-mediated ZO-1 knockdown studies in epithelial cell lines^59^. Occludin is a member of the MARVEL (MAL and related proteins for vesicle trafficking and membrane link) domain–containing family of proteins involved in membrane apposition^60^. Occludin is a tetraspan transmembrane protein that primarily localizes to bicellular tight junctions in association with binding to ZO-1^61,62^. While occludin’s importance to barrier function is somewhat controversial, it has been confirmed to contribute to tight junction stabilization and optimal barrier function by restricting macromolecule permeability and modification of claudin-2-mediated cation permeability^61^. It therefore appears to act primarily as a regulator of tight junctions rather than as an essential structural component^58^. However, conditions of cellular stress, such as inflammatory cytokine challenge, can induce internalization of ZO-1 and occludin and this action is associated with a compromised epithelial barrier^63,64^. Here, we investigated if simulated microgravity, generated by culturing IECs in the RWV, altered junction formation and functional barrier properties of polarized epithelial monolayers, and if subsequent exposure to acetaldehyde induced a differential effect on barrier integrity in cells exposed to simulated microgravity conditions. Moreover, our study is the first to investigate if functional changes to epithelial cell barrier properties were sustained over time following removal from the simulated microgravity environment.

## Results

### Growth of IECs on microcarrier beads

**Figure 1:**
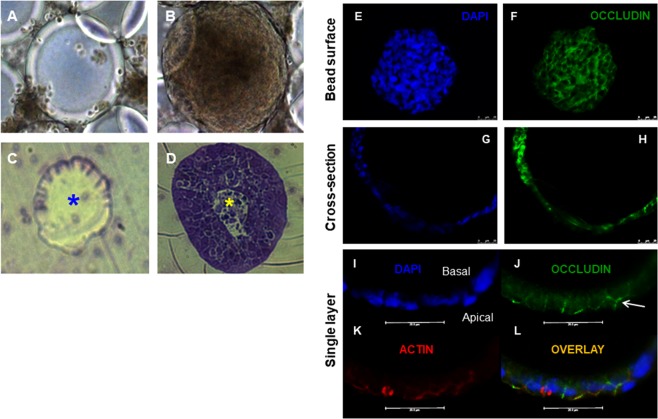
HT-29.cl19a cells attach and form 3-D monolayers on individual microcarrier beads. HT-29.cl19a intestinal epithelial cells were cultured with microcarrier beads for 18 days under the conditions of simulated microgravity in a rotating wall vessel (RWV) at 16–17.8 rpm. (**A**,**B**) Samples were removed from the RWV for observation under an inverted light microscope (Panel A,: day 1, panel B, day 11) and embedded in LR White and stained with Toluidine Blue for EM imaging (Panels C,D, day 1, day 18). Panels A and C show a bead lacking cell attachment. By comparison, panels B and D show that cells attached and grew around individual beads. (**D**) Cells in panel D were able to take up the vital dye, Toluidene Blue, thus demonstrating viability of cells *in situ* on the microcarrier bead. Panels C,D shown in cross-section with the center of the hollow bead indicated by an asterisk. (**E**,**F**) whole cell-bead aggregates were stained for nuclei (DAPI) and the tight junction protein, occludin, showing an intact layer around the surface of the bead and tight junction formation. (**G**,**H**) Cross-section shows that cells grew as a unicellular layer around the outer surface of the bead. (**I**) Staining of cellular junction formation on cell-bead aggregates shows apico-lateral staining of (**J**) occludin and (**K**) actin with (**I**) nuclei stained by DAPI and (**L**) showing an overlay image of all three stains. Arrow indicates occludin staining at a cell-cell junction.

HT-29.cl19a cells were grown on beads for 18 days in a rotating wall vessel (RWV) as previously shown^11,17,18^. Under these conditions, HT-29.cl19a cells form a single layer of cells covering each bead as demonstrated successfully in prior studies^17^. In Fig. 1 we show (A) an uncovered microcarrier bead and (B) a bead around which HT-29.cl19a cells have formed a continuous layer. Figure 1C,D show an uncovered bead and a covered bead respectively that have been embedded in LR White, sectioned and stained with the vital dye, Toluidene Blue^65^. Figure 1 D shows uptake of Toluidene Blue by cells surrounding the bead indicating that the cells are viable. We also showed that these cells grow as a monolayer around the surface of the bead. This was demonstrated by immunofluorescence staining of nuclei and the transmembrane tight junction protein occludin in whole mount imaging of beads and in cross-section (Fig. 1E–H). Furthermore, these monolayers are capable of forming tight junctions as shown by the apico-lateral membrane staining of occludin (Fig. 1I,J)^17,51,66,67^. The cytoskeletal protein, actin, that plays a critical role in maintaining the architecture of cell junctions, also localized intensely at the apicolateral membrane thus suggesting that polarization is not disrupted (Fig. 1K,L). These data show that HT-29.cl19a IECs are capable of attaching to and growing on microcarrier beads in a manner that facilitates formation of tight junctions.

### RWV culture affects barrier formation

**Figure 2:**
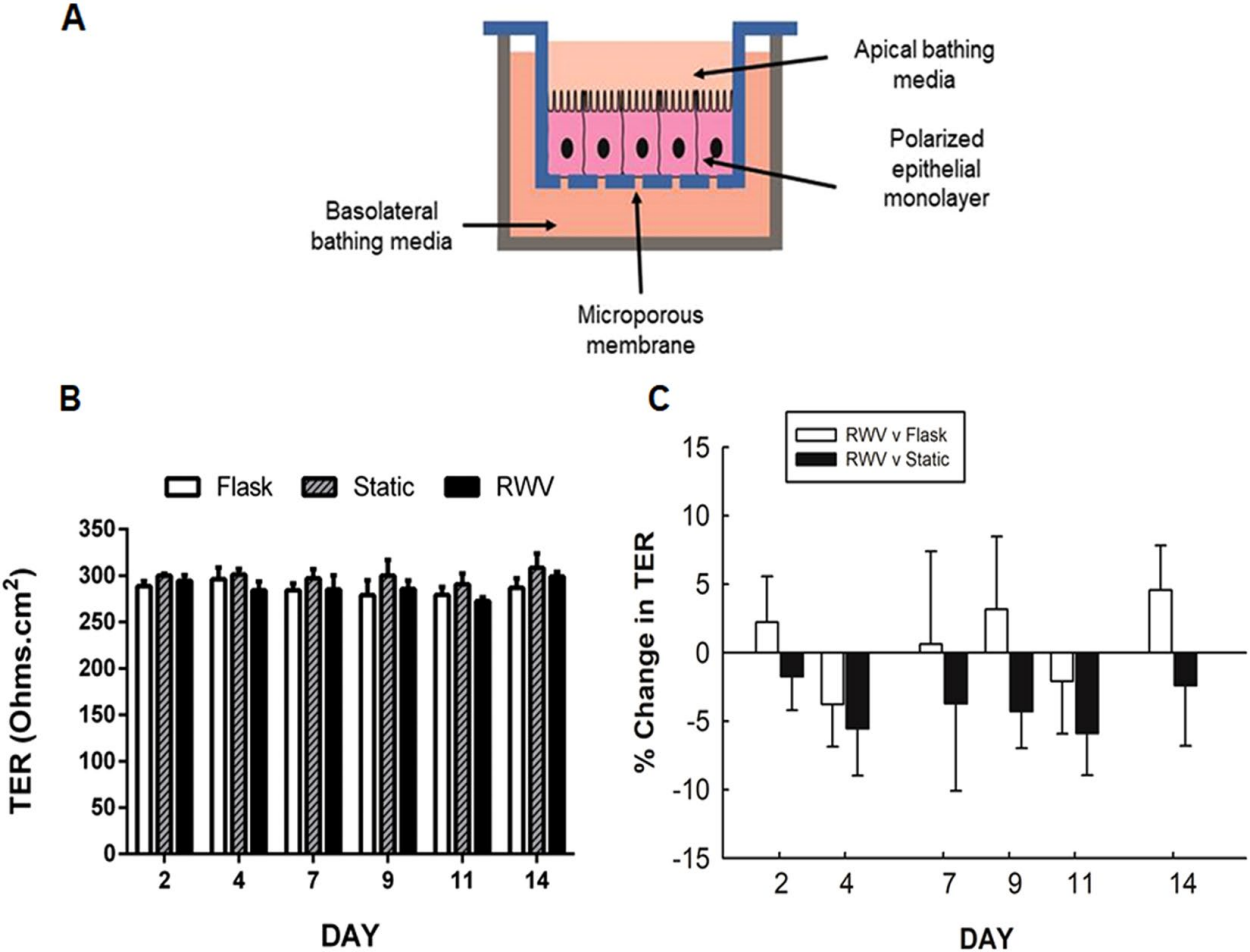
Low shear simulated microgravity exposure affects epithelial barrier formation. **A**) Model of polarized intestinal epithelial monolayers cultured on a semi-permeable support. (**B**) TER values (days 2–14) of HT-29.cl19a cells grown on semi-permeable inserts following culture under normal cell culture conditions (flask control), cultured on microcarrier beads alone (static control), or cultured on microcarrier beads and exposed to simulated microgravity (RWV). No statistically significant underlying differences in overall TER raw values were observed between conditions. (**C**) Data are shown as the % change in TER of cells exposed to microgravity *vs*. static (black) or flask controls (white). No statistically significant difference was observed in % change between the different culture conditions, although the % change in TER of cells from RWV relative to the static control was consistently lower (n = 4).

Having shown that we could culture IECs on microcarrier beads in the RWV and observe expression of tight junction proteins, we next wanted to perform functional measures of barrier integrity. In order to accomplish this however, we had to resort to growing cells on conventional inserts i.e. a 2–3D format having first exposed them to a simulated microgravity environment by culturing them on microcarrier beads (Fig. 2A). After culture in the RWV for 18 days, cells were removed from microcarrier beads by trypsinization, and seeded onto semi-permeable filter supports at a density of 5 × 10^5^ cells per support. Two separate control cultures were also prepared. A ‘flask’ control was prepared by culturing cells in a T75 flask prior to seeding on filter supports while a ‘static’ control involved culturing cells on microcarrier beads in a culture dish as opposed to the RWV. This control was particularly important as it served as the substrate control for cells in the RWV cultured on the same type of bead. Cells were cultured on inserts up to 14 days and TER was monitored over time. No significant difference was observed in raw TER values over time between the different conditions (Fig. 2B). When the relative change in TER between cells cultured in the RWV and the respective controls was calculated, there was no consistent difference detected *vs*. cells from a flask not subjected to the simulated microgravity environment (Fig. 2C). However, when cells from the RWV were compared with the appropriate substrate control – the static control – the simulated microgravity environment caused a slight but consistent drop in TER over the duration of the experiment (Fig. 2C). The most prominent change in %TER in RWV cells *vs*. static controls was observed on day 11(−6 ± 3% decrease *vs*. static; n = 4). This decrease did not reach statistical significance and was not due to differences in seeding density (data not shown). These data suggest that exposure to a simulated microgravity environment induces a subtle change in IEC barrier formation that is retained up to 14 days following removal from the simulated microgravity environment.

### Simulated microgravity alters tight junction protein localization not expression

**Figure 3:**
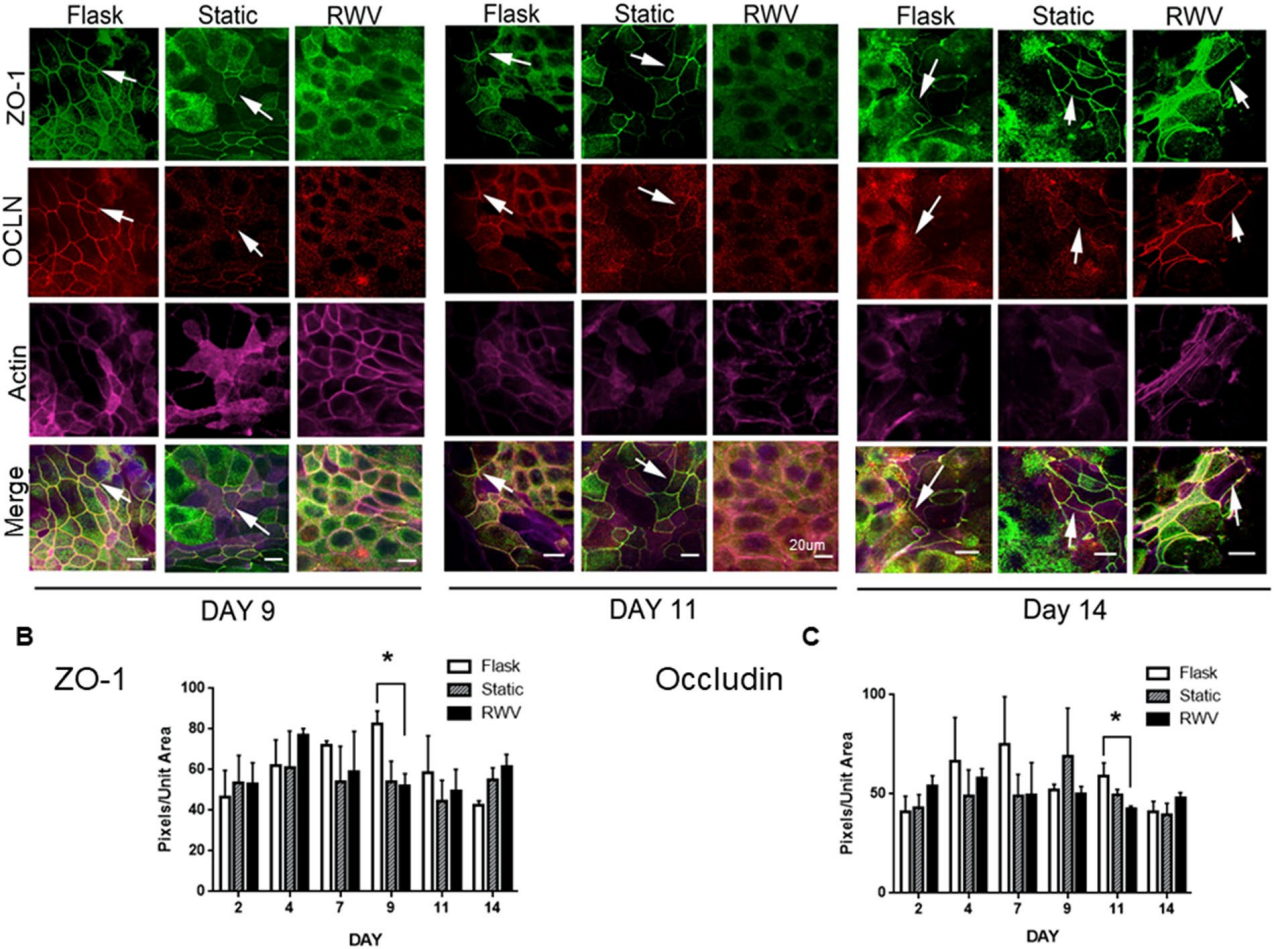
Delayed tight junction formation in IECs exposed to simulated microgravity. HT-29.cl19a IECs were fixed with 4% paraformaldehyde, immunostained for ZO-1 (green) and occludin (red) and imaged by confocal microscopy. Actin was stained with Phalloidin. Arrows indicate the presence of apical tight junctions. Reduced levels of ZO-1 and occludin were observed in apical tight junctions on day 9 and 11 in IECs exposed to simulated microgravity. Levels appeared to recover by day 14 although substantial levels of ZO-1 appeared to be present in the cytoplasm and not at the tight junction. (**B**,**C**) Confocal imaging and quantification of total B) ZO-1 and (**C**) occludin levels was performed using a Zeiss Axioscope 2 upright microscope and Zeiss LSM 5 Image Examiner software respectively. No consistent differences in protein levels were evident between the different conditions over the culture period on semi-permeable supports, although ZO-1 levels were lower in RWV and static controls *vs*. flask control at day 9, while occludin levels were reduced in the RWV condition at day 11 (*p < 0.05; n = 4).

To understand the molecular changes that might contribute to altered barrier function in cells subjected to simulated microgravity, we investigated whether cells cultured in the RWV exhibited changes in expression or localization of the key tight junction proteins, occludin and ZO-1. Localization of occludin and ZO-1 was monitored from day 9–14 post seeding on inserts after removal of cells from beads (RWV, static control) or flasks (flask control). Cells were stained *in situ* on inserts. On day 9, both ZO-1 and occludin localized to the cell membranes in flask control cells, and to a lesser extent in static control cells, as demonstrated by the sharp chicken-wire pattern of staining (Fig. 3A). However, in RWV-cultured cells, both tight junction proteins had a more diffuse sub-membranous localization. This did not appear to be due to a defect in actin localization or expression as actin showed a very clear pattern of staining in RWV-cultured cells similar to flask controls and superior to static controls. The same pattern of diffuse sub-membrane localization of ZO-1 and occludin was observed in RWV cells on day 11. However, by day 14 both ZO-1 and occludin had localized at cell membranes similar to flask and static control cultures. We quantified the staining intensity of each TJ protein over 6 intervals from day 2 to day 14 post-seeding (Fig. 3B,C). A significant decrease in ZO-1 was observed in RWV-treated cells only at day 9 and this subsequently reached equivalent expression with the flask control cells by day 11 (p < 0.05; n = 4; Fig. 3C). A decrease in ZO-1 staining was also observed in the static condition at day 9 but this did not reach significance. While occludin levels were higher in RWV cells at day 2 compared with flask and static controls, they were decreased at day 11 compared with flask controls but reached equivalent levels to both flask and static controls on day 14 (p < 0.05; n = 4; Fig. 3C). Overall, these data suggest that the altered TER in RWV cells is likely due to delayed membrane localization of ZO-1 and occludin rather than reduced expression. This may be due to possible effects on the trafficking of tight junction proteins to the apical membrane and delayed junction formation in IECs subjected to simulated microgravity.

### Simulated microgravity increases epithelial barrier susceptibility to challenge

**Figure 4:**
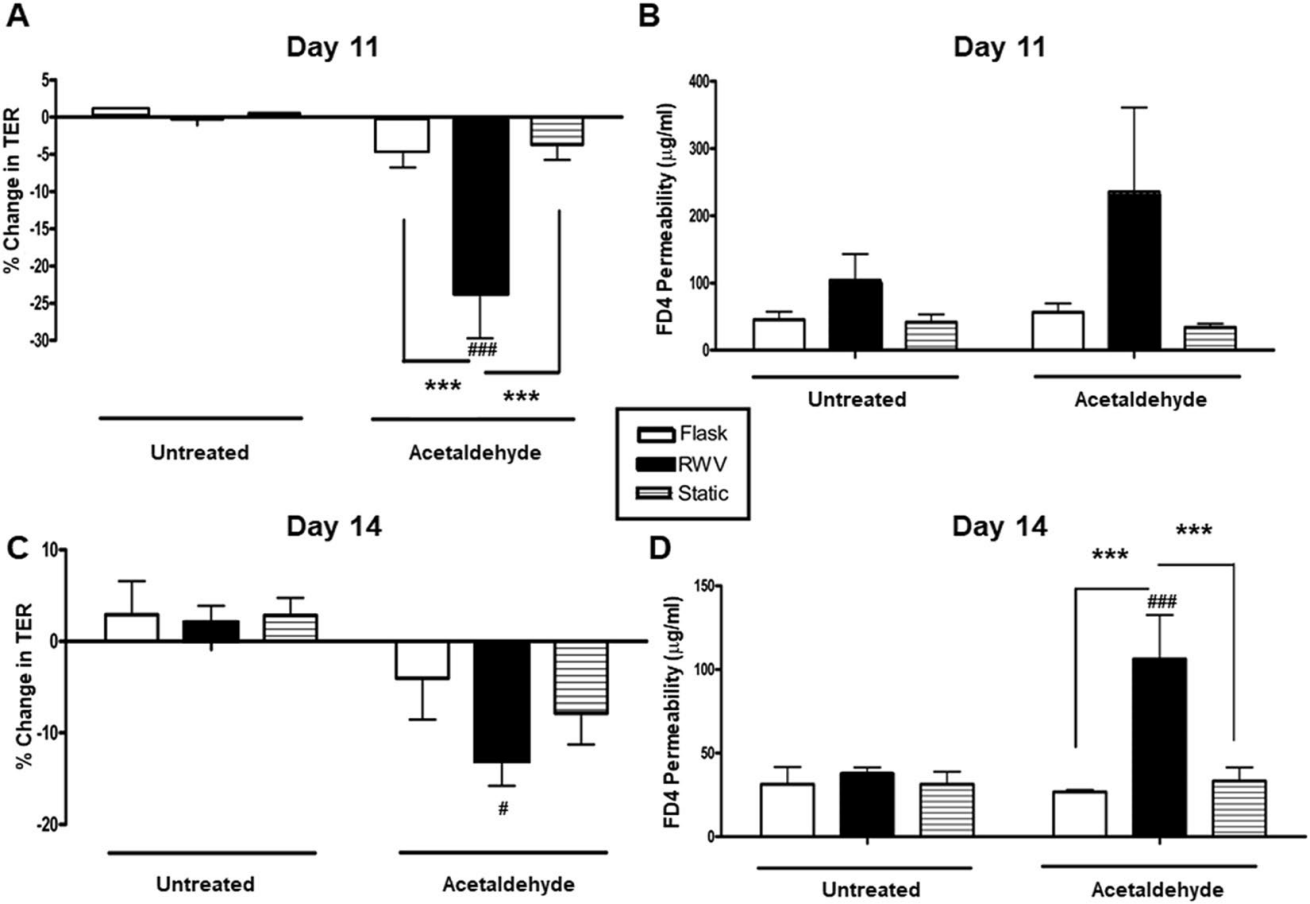
Simulated microgravity increases susceptibility of IECs to acetaldehyde-induced barrier dysfunction. (**A**) IECs grown on inserts for 11 days post-RWV culture exhibit a significant decrease in TER after 5hrs of acetaldehyde treatment (^###^p < 0.001 *vs*. RWV untreated) *vs*. static (***p < 0.001) and flask controls (***p < 0.001; n = 7). (**B**) IECs at 11-day post-RWV also showed an increase in FD4 permeability after acetaldehyde treatment *vs*. static and flask controls (n = 7). Similarly, panel C shows IECs at 14-day post-RWV exhibited a significant decrease in TER (*p < 0.05, n = 6) *vs*. untreated controls, and a non-significant decrease in TER *vs*. static and flask controls (n = 6). (**D**) IECs at 14-day post-RWV have increased permeability to FD4 in response to acetaldehyde treatment (^###^p < 0.001 *vs*. RWV untreated) *vs*. static and flask controls (***p < 0.001, n = 7).

Having shown that culturing IECs in the RWV led to a small drop in TER compared with static controls (c.f. Figure 2), we next investigated if RWV culture pre-disposed IECs to a greater drop in barrier function in response to an agent that compromises barrier integrity. Acetaldehyde is the major metabolite of alcohol and has been shown to directly reduce barrier function following *in vitro* administration to IEC cultures, as well as mediate the effects of ethanol administered *in vivo*^43,44,51^. Acetaldehyde vapor was the preferred mode of administration as it delivers a consistent and titratable dose of acetaldehyde to cells cultured in neighboring wells^68^. We treated cells at day 11 and day 14 post-RWV culture in order to assess whether barrier defects in response to challenge were apparent before and after appropriate localization of ZO-1 and occludin to cell membranes in RWV cultured cells (c.f. Figure 3). Exposure of cells on day 11 post-seeding to 0.5% acetaldehyde vapor for 5 hrs led to a significant decrease in TER in RWV cultures compared with flask and control cells (p < 0.001; n = 7; Fig. 4A). No change in TER occurred in untreated cultures over the 5 hr incubation period. In parallel, a significant increase in permeability to 4kD FITC-dextran occurred in RWV cells exposed to acetaldehyde (p < 0.001; n = 7; Fig. 4B). When cells at day 14 post-seeding were exposed to acetaldehyde, similar defects in barrier function were observed as TER was significantly reduced (p < 0.001; n = 6; Fig. 4C) while permeability to FITC-dextran was significantly increased (p < 0.001; n = 7; Fig. 4D). These data indicate that cells cultured in the RWV exhibit a significantly greater susceptibility to an agent that compromises barrier function than control cultures even when tight junction proteins appear to have localized appropriately at the cell membrane (day 14, c.f. Figure 3). Interestingly, FITC-dextran permeability in untreated RWV cells was elevated above the respective controls at day 11 but not day 14 and is thus consistent with data in Fig. 3 indicating maturation of tight junctions in cells cultured in the RWV and subsequently grown on inserts for 14 days.

### Random motion does not reproduce the effects of simulated microgravity on alcohol-induced barrier defects

**Figure 5:**
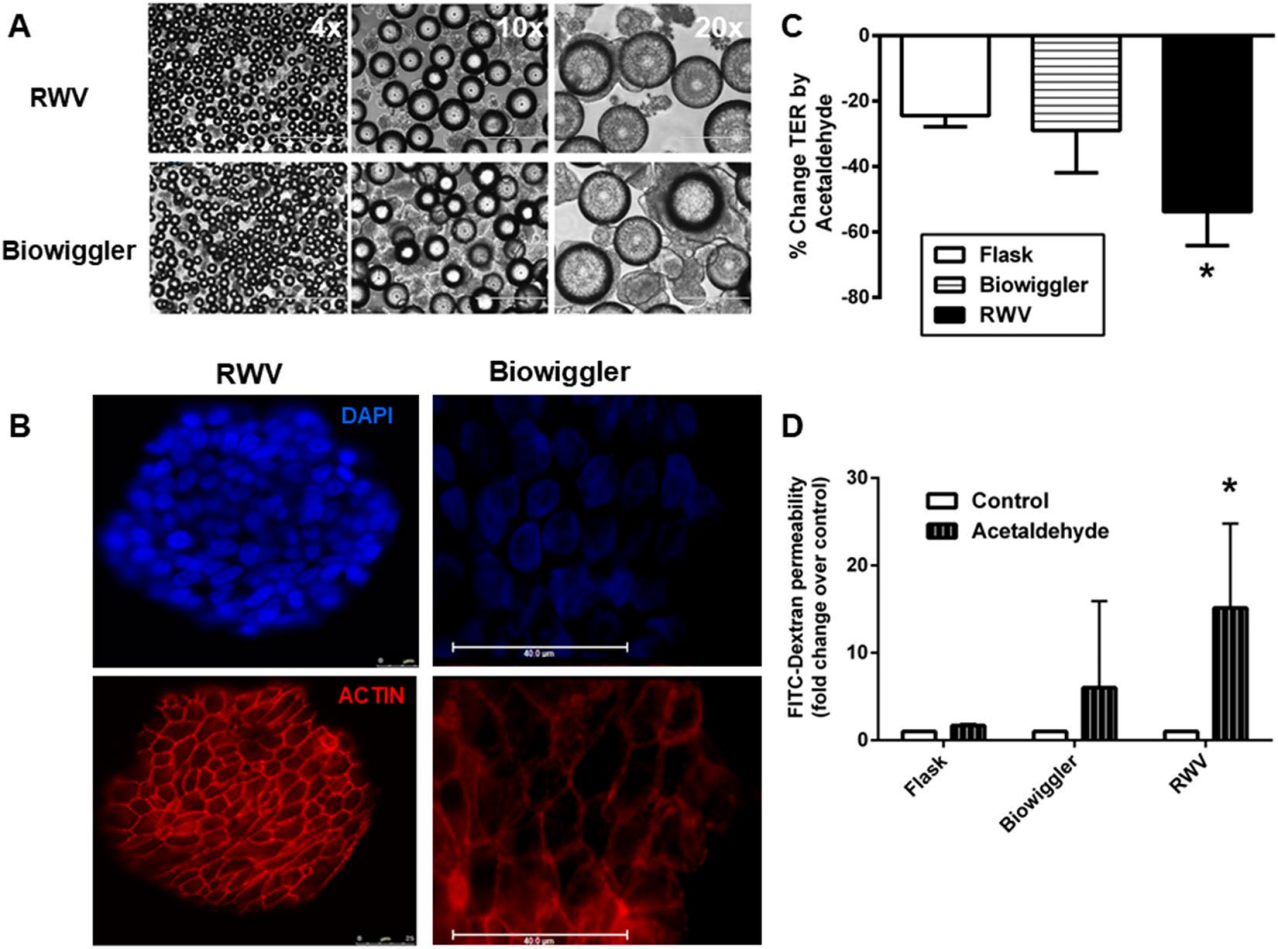
Susceptibility to acetaldehyde induction of barrier defects is specific to the simulated microgravity condition. (**A**) HT-29.cl19a IECs were cultured as microcarrier bead-cell aggregates under established RWV conditions or in a ‘Biowiggler’. Cell-bead aggregate formation was sampled at day 12 and visualized under phase-contrast microscopy. (**B**) Immunofluorescence imaging shows bead coverage by IECs and formation of cell junctions as shown by actin staining (red). Nuclei were stained with DAPI (blue). (**C**) Cells were removed from beads or a control flask and cultured on semi-permeable supports (transwells) for 11 days. TER was recorded immediately prior to exposure of cells from each condition to acetaldehyde vapor (5hrs), and the change in TER caused by acetaldehyde was calculated and expressed as the % change in TER from t0 to t5hr. Cells originally cultured in the RWV, but not the Biowiggler, showed a significantly greater decrease in TER from control flask conditions (*p < 0.05 *vs*. Flask + acetaldehyde condition; n = 3). (**D**) After 5 hrs of acetaldehyde vapor exposure, permeability to FD4 (over 1 hr) was measured and found to be significantly elevated in cells from the RWV, but not the Biowiggler compared with control cells. Data are expressed as fold change in FD4 permeability (*p < 0.05 *vs*. Flask + acetaldehyde condition; n = 3).

While the static condition served as a valuable substrate control for cells bound to microcarrier beads, we next determined if physical motion could recapitulate the effects of simulated microgravity in promoting a sustained susceptibility to barrier defects. As a model of physical motion, we cultured cell-bead aggregates in a “Biowiggler” device that can physically thrust structures in solution in 3-dimensions, with reduced unidirectional shear, at adjustable speeds to accommodate cell growth and therefore increased mass of the cell-bead aggregate over time. The Biowiggler has been utilized with a number of different cell culture systems to model the impact of motion on biological properties. We initially performed careful optimization protocols to determine the appropriate duration, speed and vigor of movement in which cell-bead aggregates could be cultured to ensure complete cell coverage of beads and subsequent successful seeding onto transwells (data not shown). HT-29.cl19a IECs were cultured on microcarrier beads for 12 days in the Biowiggler with matching cell-bead aggregates cultured in the RWV until confluent cell-bead aggregates formed (Fig. 5A). Staining for actin indicated appropriate cell cytoskeletal architecture in cell-bead aggregates cultured in the Biowiggler and RWV environments (Fig. 5B). Cells were subsequently disassociated from the beads by gentle detachment using Accutase cell detachment solution and seeded at a density of 0.5 × 10^6^ on 12 mm transwells. Cells were cultured for 11 days before exposure to acetaldehyde vapor as previously. Paired cultures were then treated with vehicle control or acetaldehyde vapor (0.5% for 5 hrs) and barrier properties were determined. TER was significantly reduced by acetaldehyde in cultures originating in the RWV compared with the control flask condition as previously (p < 0.05; n = 3; Fig. 5C). However, cells cultured in the Biowiggler showed no significant increase in susceptibility to acetaldehyde compared with control (Fig. 5C). Similarly, the acetaldehyde induction of permeability to FD4 was potentiated in cells previously cultured in the RWV compared with control (p < 0.05; n = 3), but cells cultured in the Biowiggler showed no significant increase in permeability relative to the flask control (Fig. 5D). These data indicate that the sustained increase in susceptibility to acetaldehyde challenge that occurs in cells cultured in a simulated microgravity cannot be recapitulated by randomized motion.

### Expression of epithelial tight junction proteins is unaltered by simulated microgravity with or without acetaldehyde challenge

**Figure 6:**
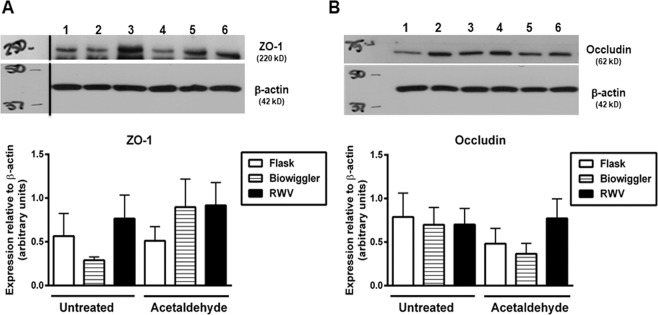
Tight junction protein expression levels are not reduced by simulated microgravity conditions. HT-29.cl19a IECs cultured in tissue culture flasks (‘Flask’), the Biowiggler or the rotating wall vessel (RWV), were grown on transwells as monolayers and subsequently subjected to 0.5% acetaldehyde vapor for 5 hours. IEC monolayers were then lysed, processed for Western blotting and probed for the proteins indicated. (**A**) Representative blots probed for ZO-1, occludin and β-actin. (**B**) Quantification of densitometric analysis from Western blot experiments performed in duplicate (*n*  =  3–4 independent experiments). Non-contiguous sections of the same blot are indicated by a black line.

Increased paracellular permeability to macromolecules such as FITC-dextran (FD4), following challenge of epithelial cells with barrier disrupting agents is often associated with reduced expression of tight junction proteins such as occludin and ZO-1. After being cultured in tissue culture flasks, the Biowiggler or the RWV, HT29 cells were subsequently seeded and grown on transwells as monolayers and subsequently subjected to 0.5% acetaldehyde vapor, as indicated. HT-29.cl19a monolayers were then lysed and processed for Western blotting. Compared to untreated HT-29.cl19a IECs previously cultured in flasks, cells grown in the Biowiggler appeared to have slightly less ZO-1 protein expression, however this was not significant (Fig. 6A; n = 3–4). Exposure to acetaldehyde of cells cultured in the Biowiggler or RWV had no effect on ZO-1 expression (Fig. 6A). Moreover, RWV culture conditions did not affect occludin expression in either the untreated or acetaldehyde vapor challenged condition (Fig. 6B). In addition, occludin expression was not significantly altered in cells from flasks or the Biowiggler exposed to acetaldehyde vapor compared to their respective untreated controls. These data suggest that cells subjected to growth conditions in the Biowiggler or the RWV, with or without subsequent exposure to acetaldehyde vapor, do not display substantial changes in the overall protein expression of ZO-1 or occludin tight junction proteins.

### Acetaldehyde alters TJ protein localization in epithelial cells exposed to simulated microgravity

**Figure 7:**
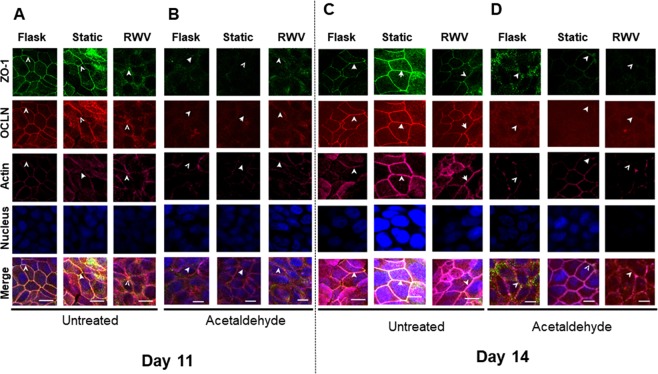
Simulated microgravity potentiates acetaldehyde-induced decrease in epithelial ZO-1. HT-29.cl19a IECs were fixed with 4% paraformaldehyde, immunostained for ZO-1 (green) and occludin (red) and imaged by Zeiss LSM 510. Actin was stained with phalloidin. Nuclear staining was performed using Hoechst 33258. Arrows indicate the position of apical tight junctions. (**A**) Reduced levels of ZO-1 were observed in apical tight junctions of IECs at 11 days post-RWV *vs*. static and flask control cells. (**B**) A greater reduction in junctional ZO-1 was observed in IECs at day 11 post-RWV following 5 hr acetaldehyde treatment versus the reduction seen in both static and flask controls. (**C**) Unchallenged IECs at day 14 post-RWV show ZO-I and occludin localization to membrane junctions similar to flask and static control conditions. (D) Acetaldehyde challenge (5 hrs) disrupted junctions in all conditions but caused greater disruption of ZO-1 and occludin staining in the day 14 post-RWV condition (n = 3).

Immunofluorescence staining for ZO-1 and occludin at day 11 showed similar findings to data in Fig. 3 where untreated RWV cells exhibited delayed localization of these proteins to the tight junctions compared with flask and static controls (Fig. 7A). All three conditions showed disrupted membrane localization of ZO-1 and occludin following 5 hr exposure to acetaldehyde vapor (Fig. 7B). Actin filaments were disrupted in untreated RWV cells on day 11, while acetaldehyde treatment disrupted the actin cytoskeletal in all three culture conditions. At day 14, when RWV cells showed normal tight junction localization of occludin, ZO-1 and an intact actin cytoskeleton in untreated conditions (Fig. 7C), RWV cells showed a greater mislocalization of ZO-1 away from the cell membrane following exposure to acetaldehyde vapor than flask or static controls (Fig. 7D). RWV and flask control cells also exhibited greater disruption of actin staining than static controls following acetaldehyde exposure. Overall, these data indicate that exposure to a simulated microgravity environment increases susceptibility of epithelial cells to acetaldehyde-induced functional barrier defects and tight junction reorganization.

## Discussion

Existing data from space flight studies indicate that a microgravity environment can have profound effects on human physiology and lead to clinical symptoms and illnesses including gastroenteritis^69^. Moreover, animal studies in spaceflight identified morphological changes in the intestine that indicated reduced jejunal villus length in rats. However, this was not attributed to defects in epithelial proliferation or migration but rather to changes in the cytoskeleton core of the intestinal villi presumably causing villus contraction^70^. To better understand the effects of microgravity on the functional behavior of the epithelial cells that line the intestinal tract, we examined the impact of simulated microgravity on intestinal epithelial cells cultured in a rotating wall vessel. The RWV generates a low shear simulated microgravity environment that enables epithelial cells to form 3-D cellular aggregates with greater *in vivo*-like characteristics, including tight junctions and extracellular processes such as microvilli, than conventional 2-D culture where cells are grown as flat, two-dimensional monolayers on impermeable surfaces^10,12,17,71^. This device has also been utilized for the purposes of creating cell cultures for tissue engineering, the study of host-pathogen interactions and drug discovery^10,12,72^. It is important to note that whereas most studies analyzing 3-D epithelial cultures have used cells grown on a flat impermeable surface as a 2-D control (flask condition in our studies), we removed cells from beads (RWV, static, Biowiggler conditions) and subsequently cultured them in a polarized manner on semi-permeable filters (transwells) in order to perform functional measurements of tight junction integrity. Therefore, these cells were grown in a more advanced system than 2-D as they are capable of correct spatial organization of apical *vs*. basolateral proteins and formation of tight junctions as determined functionally by measurement of TER, and visually by immunofluorescence staining. This necessary technical approach to facilitate functional and quantifiable measurement of barrier properties, also enabled us to determine if effects of the simulated microgravity environment in the RWV were retained by cells following removal from that environment. We demonstrated that following culture for 18 days in the RWV and subsequent seeding onto semi-permeable supports, that intestinal epithelial cells show delayed formation of tight junctions, as determined by membrane localization of occludin and ZO-1, as well as a minor reduction in barrier function. ZO-1, is a primarily apicolateral localizing protein that is critical for the formation of cell-cell junctions, although in undifferentiated cells, ZO-1 is found in other subcellular sites including the nucleus^73,74^. In our studies, ZO-1 staining in HT-29.cl19a monolayers generated from RWV-cultured cells showed a primarily cytoplasmic and perinuclear localization thus resembling ZO-1 distribution patterns in undifferentiated cells. Not until day 14 did the RWV cells display evidence of ZO-1 staining at lateral cell borders, indicative of differentiated cells. Occludin staining also exhibited delayed lateral membrane localization. This was in contrast to flask and static controls where occludin and ZO-1 had localized to the membranes as early as day 9. The delay in TJ protein localization may be due to a specific defect in trafficking of occludin and ZO-1 or possibly to an overall delay in differentiation of IEC that has been identified previously in a modeled microgravity environment^15^. In addition, the observed alterations in actin localization may contribute to a defect or delay in appropriate adherens junction protein localization, an event that precedes tight junction formation^48,75^. Indeed, the cytoskeleton has been proposed as a gravity sensor in mammalian cells that lack a specialized organelle, such as a statolith in plants, while actin is capable of acting as a mechanosensor and disruption of actin filaments has been observed in a number of cell types subjected to spaceflight microgravity or simulated microgravity^76–79^.

The significance of our data lies in the novel finding that the low shear simulated microgravity environment of the RWV imprints an altered pattern of tight junction assembly that is retained up to 14 days after removal from the RWV. Although the magnitude of the decrease in TER of RWV cells compared with static controls is modest, it is consistent with observations that although various aspects of gastrointestinal function such as transit time, mucus secretion and nutrient uptake are compromised in astronauts and rodents after time spent in spaceflight, in general they do not suffer from severe GI distress^80–84^. However, the presence of a modest underlying barrier defect may predispose to a more deleterious response to agents that can enhance barrier defects. This principle of an underlying barrier defect that is not fully manifest until exposed to challenge, underpins much research into the etiopathogenesis of a number of chronic inflammatory conditions that feature increased intestinal permeability prior to onset of inflammation. These conditions include Crohn’s disease, ulcerative colitis, celiac disease and Type 1 diabetes^32^. With this in mind, we investigated whether cells previously cultured in the RWV exhibited an increased response to an agent known to compromise barrier function, the alcohol metabolite, acetaldehyde. We tested cells grown on permeable supports at day 11 and day 14 after removal from the RWV. The significance of these time points is that evidence of tight junction assembly, as determined by tight junction protein localization, was not present at day 11 but was detectable by day 14. At day 11, we observed a clear increase in susceptibility to the effects of acetaldehyde on both TER and permeability to 4kD FITC-dextran. This was consistent with the lack of tight junction protein localization at day 11. The most striking observation was that even at day 14 when ZO-1 and occludin were localized at lateral membranes, that a significant decrease in TER and increase in FITC-dextran permeability was clearly apparent. These data indicate that in spite of appropriate TJ protein localization, that a functional barrier defect was evident following challenge with an agent that compromises epithelial permeability. We were also able to generate data indicating that the sustained susceptibility to a barrier defect (induced by acetaldehyde challenge) was not due to randomized motion but was a specific attribute imparted by the simulated microgravity environment of the RWV. Western blot studies did not show any significant change in expression of total cellular occludin or ZO-1 in unchallenged or acetaldehyde challenged 11-day old epithelial monolayers from control or RWV-cultured cells. When coupled with the immunofluorescence imaging data, this suggests that the functional barrier defects arising from RWV culture are likely mediated by changes in tight junction protein localization, and possibly signaling regulation, rather than alterations in total expression levels of individual junction proteins.

Our observations are further supported by a recent *in vivo* study using the ground-based hindlimb unloading model of microgravity simulation in mice. This identified changes in gut microbiome composition and increased susceptibility to the dextran sulfate sodium model of chemical colitis in mice subjected to hindlimb unloading^85^. The same study showed no effect of hindlimb unloading by itself on intestinal permeability as measured by *in vivo* FITC-dextran permeability. This is consistent with our findings that IECs cultured in the RWV simulated microgravity environment had no significant change in permeability until faced with experimental challenge. Moreover, while numerous studies have identified both positive and negative roles for the gut microbiome in modulating intestinal epithelial barrier function^86,87^, it is worth noting that the changes in composition and structure of the intestinal microbial community observed after 4 weeks of hindlimb unloading by Shi *et al*.^85^ were by themselves insufficient to provoke an increase in intestinal permeability. While the mice subjected to hindlimb unloading were subsequently more susceptible to challenge, the nature of the experimental challenge in this study did not selectively modify tight junction function as DSS acts by essentially destroying the epithelial monolayer thus obliterating tight junctions, whereas in our studies acetaldehyde vapor challenge of IECs does not cause chemical stripping of the epithelial lining but rather induces modulation of tight junction protein localization while keeping the monolayer intact^51,52,88^. This was confirmed in our studies by a reduction in, but not a catastrophic loss of, TER in IEC monolayers exposed to acetaldehyde.

With respect to the mechanism(s) of increased susceptibility to acetaldehyde-induced barrier disruption, Sheth *et al*.^52^ showed that acetaldehyde destabilizes the interaction of junctional proteins with the actin cytoskeleton, and this contributes to mislocalization of the tight junction proteins occludin and ZO-1. The molecular changes observed in our study are consistent with our understanding of the effects of acetaldehyde on tight junctions. The mechanisms involved in mediating this effect of simulated microgravity on barrier function and the increased susceptibility to alcohol metabolite challenge remain to be determined. They may involve epigenetic modulation of genes coding for proteins involved in junction assembly and protein trafficking, as suggested by the delayed junction localization of ZO-1 and occludin observed in cells cultured in microgravity conditions (c.f. Figure 3). These are attractive avenues for follow-up studies.

Of relevance to human health on Earth, adaptation to spaceflight shares many similarities with the effects of aging with respect to their impact on human physiology. Both of these factors exert effects at a cellular and molecular level that impact and promote a decline of multiple physiological systems in the body^89,90^. Pre- and post-flight testing has identified many physiological alterations that also occur in aging. These include bone density loss, muscle atrophy, negative calcium balance, cardiovascular changes, metabolic & endocrine effects, immune suppression and decreased gastrointestinal function^84,91,92^. One important difference between the effects of spaceflight and aging is that the effects of spaceflight are largely recoverable upon return to Earth, although flight durations have been quite short with the longest sustained period that any human has spent in space being 14 months^90^. Of note, studies of digestive dysfunction in rats during spaceflight identified that altered hepatic and xenobiotic enzyme expression persisted upon return to Earth even after other digestive parameters had alleviated^84^. Nevertheless, spaceflight and approaches that try to recreate the conditions of spaceflight including head-down bed rest in the case of humans, or devices that simulate microgravity such as the rotating wall vessel in the case of cell cultures, have been used as model systems to study the effects of aging on various physiological parameters and cell types^8,16,93,94^. A number of studies have identified increased intestinal permeability to macromolecules such as mannitol, and in particular, significantly elevated permeability in aged rodent intestine following challenge with agents that increase permeability such as phorbol ester^50,95,96^. Studies of human intestinal permeability in younger *vs*. elderly adult humans have centered on double sugar and lactulose:mannitol ratios, but while these studies did not show a clear difference between younger *vs*. older adults, interpretation of these data is complicated by an expected decrease in renal saccharide clearance with age, as well as a small sample size^97,98^. A study investigating the effects of aging on intestinal permeability in non-human primates identified an increase in macromolecule permeability to horseradish peroxidase and a decrease in the electrical integrity of aging intestine as assessed by potential difference (PD) measurements. Furthermore, these changes were also associated with decreased occludin and ZO-1 levels in colonic mucosal tissue^99^. Overall, our data share consistencies with animal studies identifying an increase in intestinal permeability associated with aging, albeit with a requirement for an experimental challenge to fully identify the nature of the barrier defect. Thus, the low-shear simulated microgravity environment generated by the RWV may also serve as a valuable model system to investigate the effects of aging on epithelial barrier function as well as other properties of epithelial cells.

The data generated using the RWV system are intriguing and although further studies into the mechanism(s) responsible for the observed barrier defect in cells exposed to simulated microgravity are needed, we can speculate on the implications of these findings. The capacity of the RWV system to induce an underlying barrier defect in cells that have appropriate tight junction localization may serve as a very useful experimental model to study the pathogenesis of disease conditions associated with underlying barrier defects prior to disease activation, such as inflammatory bowel disease, or patients with increased sensitivity to barrier-disrupting agents such as alcohol. With respect to the potential impact of a microgravity environment on the gastrointestinal tract, these data suggest that intestinal epithelial cells develop a minor barrier defect possibly without significant pathologic manifestations until such time as they are exposed to an agent, either chemical or biological, that can compromise barrier function. This could result in a greatly exaggerated barrier defect that may have dramatic consequences for epithelial integrity and intestinal homeostasis. Specifically, given previous evidence of immunosuppression and increased virulence of pathogenic bacteria in conditions of microgravity, an underlying defect in the intestinal epithelial barrier could greatly expedite bacterial colonization of the host, or effects of bacterial products, and give rise to a more rapid and severe infection. This may have particular relevance to long-term space travel and colonization where exposure to a food-borne pathogen may result in a more severe pathology than on Earth.

## Materials and Methods

### Microcarrier preparation

Cytodex-3 microcarrier beads (collagen and dextran coated, average 175 μm in size) were purchased from GE Healthcare (Piscataway, NJ) and prepared based on the manufacturer’s protocol. Beads were rehydrated in Ca^2+^ and Mg^2+^ free phosphate buffered saline (PBS)(50–100 ml/g) for 3 h at room temperature. Beads were sterilized by removal of the supernatant and 70% ethanol (70–100 ml/g) was added. Beads were washed twice in this manner and incubated in 70% ethanol solution overnight. Prior to use, beads were washed three times in PBS (50 ml/g) and once with culture medium (50 ml/g) before use. Corning enhanced attachment microcarrier beads, (cat#3779, Corning Inc., Corning, NY) were reconstituted in sterile, molecular grade water at a concentration of 200 mg/ml.

### Cell culture in the rotating wall vessel system

The development of 3-D cell culture model was performed as previously described^17,71,100^. HT-29.cl19a cells were initially grown as monolayers with fresh McCoy’s 5A media with L-glutamine (Mediatech, Manassas, VA) supplemented with 10% fetal calf serum (Hyclone, Waltham, MA; catalog # SH3007303), Penicillin (500 I.U.) and Streptomycin (10,000 ug/ml) (Mediatech) culture medium in T-75 cell culture flasks (Corning, Inc. Corning, NY) at 37 °C in a 5% CO_2_ environment in preparation for seeding into the rotating wall vessel (STLV, Synthecon, Inc, Houston, TX). Cells were cultured until approximately 70–90% confluent. HT-29.cl19a monolayers were washed once with 0.25% Trypsin-ethylenediamine tetraacetic acid (Gibco, Carlsbad, CA), removed from flasks by treatment with 0.25% Trypsin-EDTA for 15 minutes at 37 °C and resuspended in fresh culture medium, supplemented with FBS (5%) and L-glutamine (2.5 mM), at a density of 2–2.2 × 10^5^ cells/ml. Cells were assayed for viability by trypan blue exclusion. Cells were introduced to the RWV containing 5 ml/mg of Cytodex-3 microcarrier beads, or 250 mg of Corning microcarrier beads, resulting in a ratio of approximately 10 cells/bead. Cells were cultured in the RWV at a rotation speed adjusted to maintain beads and cells in suspension (16.8–17.8 rpm). HT-29.cl19a cells cultured with beads in a petri-dish in the absence of rotation exposure served as a substrate (Static) control. Fresh medium was replenished by 80–90% of the total volume after 3 days initially and every 24–48 h thereafter.

### Cell culture in the random motion incubator (Biowiggler)

The Biowiggler^TM^ is a PC programmable eight-position stirrer that simplifies cell culture applications (Global Cell Solutions, Charlottesville, VA). Each of eight magnetic drive positions can be independently programmed to stir intermittently, continuously or “wiggle” in a reversing two-way motion. Proprietary LeviTubes (50 mL volume) were preconditioned for 24 hrs prior to cell seeding by adding warm PBS (1×) and placed in the Biowiggler to rotate bidirectionally at 40 rpm inside the CO_2_ incubator. Phosphate buffer was replaced by 25 mL of pre-warmed McCoys 5A media enriched with 10% FBS. To the LeviTubes and in sequence, 100 mg of reconstituted Corning enhanced attachment microcarrier beads (Corning, cat#3779; Corning Inc., Corning, NY) were added, followed by 5 × 10^6^ HT-29.cl19a cells to a final volume of 40 mL. LeviTubes were placed in the CO_2_ incubator without shaking to allow the cells to attach to the carrier beads for 5 minutes. For the first 24 hours, LeviTubes were rotated bidirectionally at 40 rpm followed by a 5-minute static rest period. This combined period allowed rounded cells make contact with carrier beads (adsorption), followed by flattening of cells on the side of contact (attachment) to promote cell attachment to the microcarriers. Thereafter, cell aggregates continued to grow and proliferate by continued bidirectional rotation at 40 rpm. Media and cell cultures (50 μl aliquots) were monitored every 2 days. Media was changed every day.

### Harvesting cells from microcarrier beads

Cells were harvested 18 days after initial seeding in the RWV or in respective control environments (Biowiggler; culture flask). Cells cultured with beads were removed from the RWV or petri-dish, allowed to settle, and incubated with Ca^2+^ and Mg^2+^ PBS containing 0.02% EDTA (w/v) (50–100 ml/g beads, pH7.6) for 5 minutes at 37 °C. The PBS-EDTA was removed and the beads were incubated with Accutase cell detachment solution (Corning^TM^ cat 25058CI, Corning, NY) at 37 °C with occasional agitation for 15 minutes. After 15 minutes, 30 ml culture medium was added and detached cells separated from beads by filtration through a 100μm nylon filter (Millipore, Bellerica, MA). Detached HT-29.cl19a cells were re-suspended at a density of 2.5 × 10^5^ cells/ml and seeded onto 12 mm semi-permeable polycarbonate inserts (Millipore) in 24-well plates for 9,11 or 14 days for functional measurements of barrier integrity and to assess junction assembly.

### Cell culture conditions for barrier function assays

Four cell culture growth conditions were used for seeding and growth of epithelial monolayers on transwells for the measurement of barrier properties:

#### Flask control

Monolayer controls for HT-29.cl19a were grown in T-75 flasks for 10–18 days prior to seeding on transwell inserts.

#### RWV

As described in detail above, HT-29.cl19a cells were cultured in the RWV at a density of 2.0–2.2 × 10^5^ cells/ml with microcarrier beads in a 10:1 ratio (cells:beads) at a rotation speed adjusted to maintain beads and cells in suspension (16.8–17.8 rpm) for 18 days.

#### Static control

HT-29.cl19a cells cultured at a density of 2.0–2.2 × 10^5^ cells/ml with microcarrier beads in a 10:1 ratio (cells:beads) in a petri-dish in the absence of rotation exposure. Media changes were performed to coincide with those of the RWV. This condition served as a microcarrier bead substrate control without the effects of the RWV (Static) control.

#### Biowiggler

As described in detail above, 5 × 10^6^ HT-29.cl19a cells in cell culture media were added to custom-specific Levi tubes for pre-attachment prior to defined adsorption and attachment to microcarrier bead phases. Cell aggregates continued to grow and proliferate by continued bidirectional rotation at 40 rpm. Media and cell cultures were monitored every two days up to 18 days.

### Acetaldehyde treatment studies

Acetaldehyde (200–400 μM)(Sigma Chemical Co, St. Louis, MO), treatment was performed as previously described^51,68^. After 11 or 14 days of culture post- seeding on transmembrane inserts, HT29Cl.19a cells were preincubated with PBS containing 1.33 mM CaCl_2_ and 1 mM MgCl_2_ for 1 h. Cells were then exposed to 0.5% vapor-phase acetaldehyde in adjacent wells in a sealed 24-well culture plate for 5 h^51^. Cyanamide (10–50 μM) was added apically to inserts at the start of treatment to inhibit aldehyde dehydrogenase^51^. Addition of 0.5% vapor-phase acetaldehyde in the adjacent wells results in an approximate 400 μM acetaldehyde concentration in the buffer incubating the cell monolayers^51^.

### Transepithelial permeability studies

For permeability studies, HT-29.cl19a cells grown on semi-permeable membranes for 11 and 14 days were incubated with 1 mg/ml FITC-dextran in the apical well. At the end of acetaldehyde treatment, 100ul of media from the basolateral well was removed and fluorescence was measured with a microplate reader (SpectraMax M2, Molecular Devices). Transepithelial permeability was measured as 4-kilodalton fluorescein isothiocyanate-dextran (FITC-dextran; FD4) (Sigma Chemical Co, St. Louis, MO) flux across IEC monolayers.

### Transepithelial electrical resistance

After 18 days of culture in the presence or absence of simulated microgravity, HT29.cl19a cells were seeded onto 12 mm semi-permeable inserts and cultured for 11 or 14 days. The transepithelial electrical resistance (TER) across HT-29.cl19a monolayers was assessed before and after treatment using a voltohmeter (WPI, Sarasota, FL) and electrodes (WPI). To normalize the variation in TER, data were expressed as percentage of pre-treatment TER values.

### Immunofluorescence microscopy

2.5 × 10^5^ HT-29.cl19a cells were seeded onto 12 mm Millicell-HA filters for 11 and 14 days prior to acetaldehyde treatment. After treatment, monolayers were fixed with 4% paraformaldehyde in PBS for 10 minutes at room temperature, permeabilized with 0.1% Triton-X 100 in PBS for 10 minutes, and quenched with 50 mM ammonium chloride for 5 minutes. Between steps, cells were washed (x3) with PBS. Blocking was performed with 1% bovine serum albumin in PBS at 4 °C overnight. Cells were incubated with primary antibodies overnight at 4 °C in a humidified chamber. After PBS washing (x3), secondary antibodies: Alexa-488 conjugated goat anti-mouse (excitation/emission maxima at 495/519 nm; Molecular Probes, Eugene, OR), Alexa-568 conjugated goat anti-rabbit (excitation/emission maxima at 578/603;Molecular Probes, Eugene, OR) were added at a 1:100 dilution, and Alexa-647 Phalloidin (excitation/emission maxima at 650/668 nm; Molecular Probes, Eugene, OR) was added at 1:25 dilution, in 1% BSA in PBS for 1 hour at 37 °C. After washing (three times with PBS), cells were incubated with Hoechst 33258 (excitation/emission maxima at 352/461 nm; Molecular Probes) in PBS (1:100) for 5 min at 37 °C. The final washing stage consisted of 3 washes with PBS. Then the cells on the filter membrane were transferred onto glass slides and mounted in ProLong Gold Antifade Reagent (Molecular Probes). Confocal microscopy was performed using a Zeiss LSM 510 laser-scanning confocal system on a Zeiss Axioscope 2 upright microscope (Zeiss, Jena, Germany). Quantification of confocal images was performed using ImageJ. Briefly, the multicolor confocal image was separated into its composite colors using the Zeiss LSM 5 image examiner software. The resulting single-color field was exported into ImageJ and mean fluorescence intensity (MFI) was calculated using the Analyze-Measure function. MFI was measured and averaged from 5 fields/replicate from 3–4 independent experiments.

Microcarrier bead-cell aggregates were fixed in 4% buffered PFA, washed in PBS (Mg^2+^/Ca^2+^) and embedded in O.C.T. (VWR). 5–8 μm sections were mounted on microscope slides, washed with PBS (Mg^2+^/Ca^2+^), permeabilized with 0.3% Triton in PBS for 30 minutes and consecutively blocked using 10% normal donkey serum in PBS-T (0.02%). Cells were stained using occludin primary antibody (Life Technologies) and after PBS wash, incubated with Alexa-488 conjugated anti-rabbit secondary antibody and Alexa-647 Phalloidin (Jackson ImmunoResearch Labs, West Grove, PA.) Cells were mounted using ProLong® Gold Antifade Reagent with DAPI (Life Technologies, Carlsbad, CA). Bead-cell aggregates were visualized under phase-contrast microscopy (EVOS microscope; AMG/Thermofisher, Waltham, MA). Immunofluorescence staining of bead-cell aggregates were imaged using a Leica DM5500 fluorescent microscope (Leica Microsystems, Inc., Buffalo Grove, IL). Image panels were assembled into complete figures in Adobe Photoshop (San Jose, CA).

### Western blotting

After acetaldehyde treatment, cells from inserts containing HT-29.cl19a monolayers were suspended in ice-cold lysis buffer (50 mM Tris, 150 mM NaCl, 0.1% SDS, 0.5% sodium deoxycholate, 20 µM NaF, 1 mM EDTA, 1 µg/ml antipain, 1 µg/ml pepstatin, 1 µg/ml leupeptin, 1 mM NaVO_3_, 100 µg/ml phenylmethylsulfonyl fluoride). Cells were mechanically detached followed by centrifugation at 10,000 rpm for 10 minutes. Cell lysate supernatants were assayed for protein content using a Bio-Rad protein assay kit (Bio-Rad, Hercules, CA). Equal concentrations of proteins lysates were resuspended in loading buffer (60 mM Tris-Cl pH 6.8, 2% SDS, 5% β-mercaptoethanol, 0.01% bromophenol blue, 10% glycerol) and incubated at 95 oC for 10 minutes. Proteins were separated by SDS-PAGE and transferred onto polyvinylidene fluoride membranes (Millipore). Membranes were blocked with nonfat milk in TBS-T (Tris-buffered saline with 0.1% Tween-20) for 1 h at room temperature prior to addition of primary antibody; ZO-1 (1:1000 dilution, #61-7300, ThermoFisher Scientific, Waltham, MA), or occludin (1:1000 dilution, #71-1500, Invitrogen, Carlsbad, CA), in 1% nonfat milk in TBST. Membranes were incubated overnight at 4 ^o^C^101^. Membranes were washed 5x for 5 min per wash with TBS-T, then incubated with peroxidase-conjugated secondary antibodies (goat anti-mouse (#115-036-062) or goat anti-rabbit (#111-036-045); Jackson Immunoresearch Laboratories, Inc. West Grove, PA) diluted at 1:5000 in 1% nonfat milk in TBST for 1 h at room temperature. Immunoreactive proteins were detected using SuperSignal West Pico chemiluminescence detection kit (Thermo Scientific, Rockford, IL). Densitometric analysis of Western blots was performed using ImageJ software (National Institutes of Health).

### Statistical analysis

Data are presented as ± SEM for a series of n experiments. Data are presented as percentage of respective controls, arbitrary units, or fluorescence units. Statistical analysis was performed by analysis of variance (ANOVA) and Bonferroni post-test using Graphpad Prism Software (GraphPad Software, La Jolla, CA). P values ≤ 0.05 were considered significant.

## Supplementary information

Supplementary Figure 1<b>
