# Supplementary material for: A Simulated Microgravity Environment Causes a Sustained Defect in Epithelial Barrier Function
Source: Sci Rep. 2019 Nov 26;9:17531. doi: 10.1038/s41598-019-53862-3 (PMC6879622; doi:10.1038/s41598-019-53862-3)
Supplement: Supplementary file 1 — Supplementary Figure 1 [file 41598_2019_53862_MOESM1_ESM.pdf]

A Simulated Microgravity Environment Causes a Sustained Defect in Epithelial Barrier Function

Rocio Alvarez[1@,], Cheryl A. Stork[1,2], Anica Sayoc-Becerra[1], Ronald R. Marchelletta[2,#], G. Kim Prisk[2,3], Declan F. McCole[1].

[1]Division of Biomedical Sciences, University of California, Riverside, Riverside, CA 92521; [2]Department of Medicine, [3]Department of Radiology, University of California, San Diego, La Jolla, CA 92093;

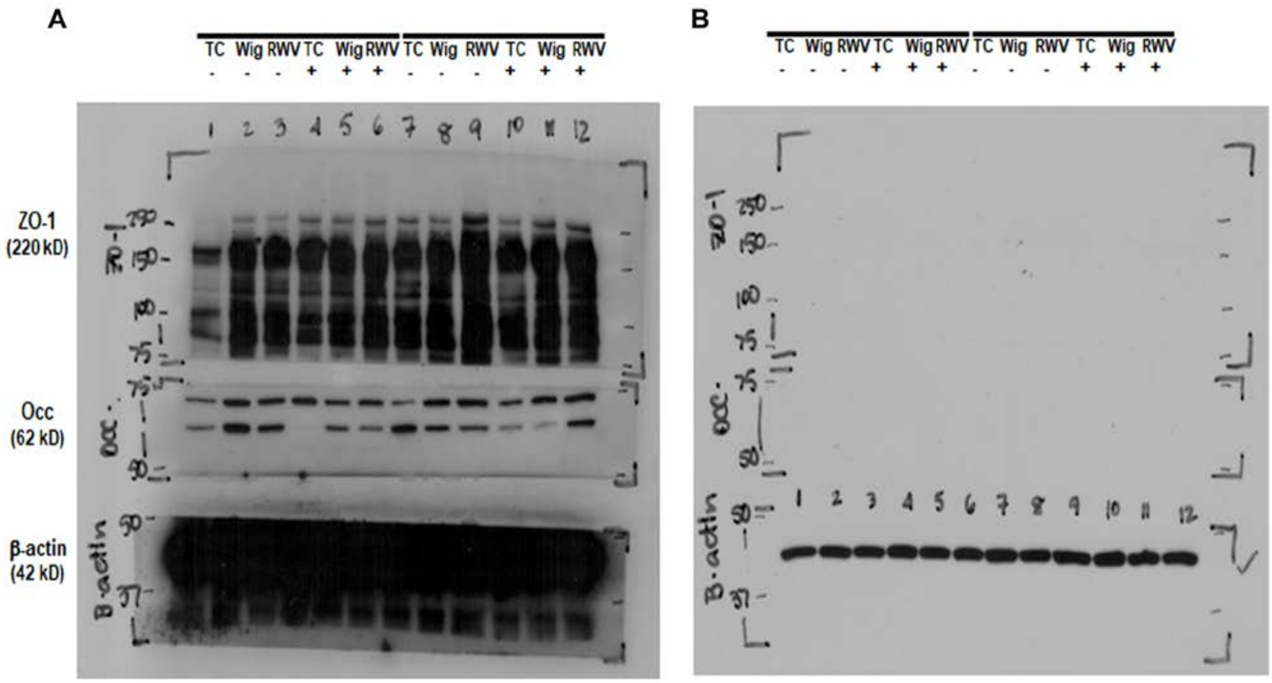

Supplementary Figure 1. Uncropped blots used in Figure 6 showing HT-29.cl19a intestinal epithelial cell expression levels of tight junction proteins, ZO-1 and occludin, as well as β-actin loading control. Blotting membranes were pre-cut and full size of the individual membranes stained for the particular protein are shown. **A.** High exposure of membranes used in Figure 6 to show cut edges of membranes. **B.** Lower exposure of β-actin blot (Suppl Figure 1; panel A) used in Figure 6 to show cut edges of membrane.
